# Endophytic Communities of Transgenic Poplar Were Determined by the Environment and Niche Rather Than by Transgenic Events

**DOI:** 10.3389/fmicb.2019.00588

**Published:** 2019-03-26

**Authors:** Yanbo Wang, Weixi Zhang, Changjun Ding, Bingyu Zhang, Qinjun Huang, Rongfeng Huang, Xiaohua Su

**Affiliations:** ^1^State Key Laboratory of Tree Genetics and Breeding, Research Institute of Forestry, Chinese Academy of Forestry, Beijing, China; ^2^Key Laboratory of Tree Breeding and Cultivation, National Forestry and Grassland Administration, Beijing, China; ^3^Institute of Biotechnology, Chinese Academy of Agricultural Sciences, Beijing, China; ^4^Co-Innovation Center for Sustainable Forestry in Southern China, Nanjing Forestry University, Nanjing, China

**Keywords:** *Populus alba* × *P. berolinensis*, endophytic communities, transgenic events, environmental condition, niche differentiation, amplicon Illumina MiSeq

## Abstract

Microbial communities associated with plants represent key determinants of plant health, survival, and growth. However, a good understanding of the structural composition of the bacterial and fungal microbiome present in different plant tissues and growing environments, especially in transgenic woody plants, is required. In the present study, we hypothesized that environmental conditions, ecological niches, and transgenic events could influence the community structure of plant-associated microorganisms (bacterial and fungal endophytes). We sampled the root and stem endospheres of field-grown transgenic and non-transgenic poplar trees (*Populus alba* × *P. berolinensis*) and applied 16S rRNA and internal transcribed spacer amplicon Illumina MiSeq sequencing to determine the bacterial and fungal communities associated with the different plant habitats and tissues. We found that actinobacteria, proteobacteria, bacteroidetes, and firmicutes were the dominant endophytic bacteria, and the fungal community was dominated by dothideomycetes, agaricomycetes, leotiomycetes, and sordariomycetes. In conclusion, transgenic events did not affect the endophytic bacterial and fungal diversity of poplar trees. The bacterial and fungal community structure depends on the pH and the soil organic matter content. Each plant tissue represents a unique ecological niche for the microbial communities. Finally, we identified the indicator operational taxonomic units (OTUs) and core microbiome associated with the different plant tissues of *Populus* and different environmental conditions. The results provide a basis for further study of host-microbial interactions with the identified abundant OTUs of *Populus*.

## Introduction

In recent years, the interaction between eukaryotes and prokaryotes has been one of the most popular areas of biological research, including the relationship between plants and microbes. Understanding the role of microorganisms during plant growth and development could allow their exploitation for human benefit.

Plant endophytic microorganisms are the communities of bacteria, archaea, fungi or viruses inhabit that plant tissues for at least a period of their life cycle, and have no negative effects on their hosts (Hallmann et al., 2011; Nerva et al., 2019). They play a key role in promoting plant growth, nutrient accumulation, and resistance to biotic and abiotic stresses, such as diseases, insect infestations, high temperature, salt, or drought (Hardoim et al., 2008; Prischl et al., 2012; Naveed et al., 2014). Studies have shown that any alteration in the diversity or activity of plant endophytic communities can have a significant impact on plant growth and environmental adaptation (Redman et al., 2011; Naveed et al., 2014; Vandenkoornhuyse et al., 2015). In addition, host genotypes might influence the microbiome that engages in symbiosis with plants. Silva et al. (2014, 2016) found that the decisive factor of changes in the endogenous bacterial, fungal, and archaeal communities of maize was the genotype of the maize plants. Meanwhile, other studies showed that endophytic bacteria from maize, rice, and potato were more susceptible to the different stages of plant development and pathogen exposure (Rasche et al., 2006; Janpen et al., 2009; Inceoğlu et al., 2010; Rangjaroen et al., 2014). Some plant endophytes colonize their hosts from the rhizosphere and move into plant roots, and are mainly defined by the soil type (Bulgarelli et al., 2012). Endophytic microorganisms also differ among different tissues of plants. Beckers et al. (2017) demonstrated that the endophytic bacterial communities in roots, stems, and leaves was highly variable compared to that of the rhizosphere, and each plant compartment represents a unique ecological niche for the bacterial communities.

Although genetically modified plants (GMPs) have provided huge economic benefits, there are still general concerns over their biosafety and ecological compatibility, including the unintended effects on the microbial communities of the rhizosphere and endosphere. Genetic modification may alter the metabolic products in plants and root secretions, and thus affect the environment for the endophytes in plant tissue and microorganisms in rhizospheric soil (Hussain et al., 2018). A study of transgenic imidazolinone-tolerant sugarcane revealed that the fungal community of the leaves was affected by the transgenic modification (Stuart et al., 2010). By contrast, a study of the endophytic bacteria in transgenic potato leaves showed no difference compared with conventional potatoes, but demonstrated changes according to the phenological stage of the potatoes (Heuer and Smalla, 1999). Sun et al. (2017) observed no significant differences in population size or dynamics of endophytic bacteria translocated from the roots of maize plants to the stem and leaves between transgenic maize with the *cry1Ah* gene and non-transgenic plants under laboratory or field conditions.

Once the transgenic plants are released into the field, especially perennial woody plants, their impact on the natural environment will be a long-term process. Therefore, the long-term study of a perennial plant is particularly important. However, to date, studies on the effects of transgenic plants on the rhizosphere and endogenous microorganisms have mainly focused on annual crops or herbaceous plants, while there are few reports on trees with long growth cycles. Previous research in our laboratory found no significant differences in bacterial communities between rhizosphere soils of 8-year-old genetically modified (GM) and non-GM poplar (*Populus* × *euramericana* ‘Guariento’) (Zhu et al., 2016).

In the present study, we evaluated the microbiome differentiation of bacterial and fungal communities associated with the root and stem endosphere of transgenic clone (A) and non-transgenic clone (B) of hybrid poplar clones (*Populus alba* × *P. berolinensis*) grown in saline and non-saline sites using 16S rRNA and internal transcribed spacer (ITS) Illumina MiSeq sequencing. Poplar (*Populus* spp.) is considered as one of the fastest growing trees, with marked economic benefits and applications in the production of biofuels, pulp, paper, and other bio-based products, such as chemicals and adhesives (Sannigrahi et al., 2010). Poplar is easily transformed and clonally propagated, and there is a wealth of genomic information available, making it the model of choice to study the biology of woody perennials, including the interaction between plants and microorganisms (Beckers et al., 2016b, 2017). In the present study, we focused on three main questions: (i) Does genetic modification change the microbial community structure in poplar? (ii) How variable are microbial communities of poplars grown in saline (Daqing) and non-saline (Qiqihar) sites? (iii) What are the differences between the microbial communities between the aboveground and underground parts of poplar?

## Materials and Methods

### Site Description and Sampling

The study was carried out at two sites in Heilongjiang province of northeast China: A saline site in Daqing (46°34′N, 125°08′E; D) and a non-saline one in Qiqihar (47°27′N, 122°51′E; Q). Both test sites are located in the Songnen Plain, which has a temperate continental monsoon climate, a mean annual temperature of 4°C, precipitation of 415 mm, and an elevation of 146 m above sea level. The trees in the experimental forests were mature and of similar age [about 10 years old, planted in 2007 (D) and 2009 (Q), respectively].

The root and stem samples of a transgenic clone (A) and non-transgenic clone (B) of hybrid poplar clones (*Populus alba* × *P. berolinensis*) were collected. The transfected exogenous gene was the *JERF*36 gene, encoding AP2/EREBP plant transcription factors, which are related to plant stress resistance. The neomycin phosphotransferase II gene (*NPT* II) derived from *Escherichia coli* transposon Tn5 was used as a marker, which provided the plants with kanamycin resistance. The transgenic poplars were obtained by agrobacterium-mediated transformation (Li et al., 2009). The trees were planted in a density of 2600 trees per hectare, with an inter-plant distance of 2 m. In each test site, three blocks were selected and within them, three transgenic trees and three non-transgenic trees were randomly selected (six trees per site). Roots (R) and soil samples were collected at a depth of 20 cm below ground level from two test sites (D and Q) in July 2015 (the peak season for plant growth in northeast China) after removing the litter layer. At the same time, annual stems (S) were also selected. In total, 24 samples were analyzed (3 × 4 root samples: DAR, DBR, QAR, QBR; 3 × 4 stem samples: DAS, DBS, QAS, QBS). Roots and stems with diameters ranging from 0.3 to 0.5 cm were selected for microbial diversity analysis. In addition, three randomly selected soil samples were analyzed from each site. The roots, stems, and soil samples were placed into an icebox and transported to the laboratory for further experiments immediately.

### Processing of Samples

To remove both epiphytic bacteria and fungi, root and stem fragments were subjected to the following process: Firstly, soil was gently separated from roots and stems by sequential washing with sterile water, and then soaked in 70% (v/v) alcohol for 2 min, and then in sodium hypochlorite solution (with 2.5% active Cl^-^) for 5 min. Subsequently, the roots and stems were washed five times with sterile water and the water removed using sterile absorbent paper (Beckers et al., 2016a). Finally, the plant samples were portioned into small fragments (2–3 cm) using a sterile scalpel and stored at -80°C until DNA extraction. All steps were performed under sterile conditions.

### DNA Extraction

Microbial DNA was extracted from 250 mg of popular roots and stems using the Power Soil DNA Isolation Kit, following the protocol provided by the manufacturer (MoBio, Carlsbad, CA, United States). The final DNA concentration and purity were assessed using a NanoDrop 2000 ultra violet-visual (UV-vis) spectrophotometer (Thermo Scientific, Wilmington, DE, United States), and the DNA quality was checked using 1% agarose gel electrophoresis.

### PCR Amplification and Illumina MiSeq Sequencing

Bacterial 16S rRNA amplicon libraries were generated via two-step PCR. DNA samples from all samples were individually amplified using a thermocycler PCR system (GeneAmp 9700, ABI, Foster City, CA, United States). Based on Bulgarelli’s optimization experiments with 16S rRNA primer pairs (Bulgarelli et al., 2015), we selected primer 799F (5′-AACMGGATTAGATACCCKG-3′), with three mismatches with the poplar chloroplast 16S rRNA, and primer 1392R (5′-ACGGGCGGTGTGTRC-3′). A first round of PCR amplification was conducted using these primers. PCR reactions were performed in triplicate with a 20-μl mixture containing 4 μl of 5 × FastPful Buffer, 2 μl of 2.5 mM dNTPs, 0.8 μl of each primer (5 μM), 0.4 μl of FastPfu Polymerase, 0.2 μl bovine serum albumin and 10 ng of template DNA. Cycling conditions included an initial denaturation at 94°C for 3 min; followed by 27 cycles of denaturation at 94°C for 30 s, annealing at 55°C for 30 s, and extension at 72°C during 45 s; and a final extension phase was performed at 72°C for 10 min. The resulting PCR products were cleared from residual primers and primer dimers by separation on a 2% agarose gel (100 V, 30 min). The target products (amplicon length = 593 bp) were excised to eliminate the mitochondrial by-products (1000 bp) and the DNA was extracted from the gel slices using an AxyPrep DNA Gel Extraction Kit (Axygen Biosciences, Union City, CA, United States) according to the manufacturer’s protocol. Next, a second round of PCR amplification was performed on the purified amplicons using primers 799F (5′-AACMGGATTAGATACCCKG-3′) and 1193R(5′-ACGTCATCCCCACCTTCC-3′) to reduce the amplicon length (394 bp) for sequencing. The reaction system and procedures were identical to the first round, except for the number of PCR cycles, which was reduced to 13.

A negative control (without DNA) was included in each PCR round to evaluate the presence of contaminating sequences in the reagents. The second round of PCR products were purified to remove residual primers and primer dimers using the AxyPrep DNA Gel Extraction Kit (Axygen Biosciences) and quantified using QuantiFluor-ST (Promega, Madison, WI, United States).

At the same time, Fungal ITS amplicon libraries were generated using specific primers ITS1F (5′-CTTGGTCATTTAGAGGAAGTAA-3′) and ITS2R (5′-GCTGCGTTCTTCATCGATGC-3′). The reaction system, purification, and quantitative procedures were identical to the first round of bacterial PCR.

Finally, purified amplicons were pooled in equimolar amounts and paired-end sequenced [(2 × 250 bp) for Bacteria, (2 × 300 bp) for Fungi] on an Illumina MiSeq platform (Illumina, San Diego, CA, United States) according to standard protocols by Majorbio Bio-Pharm Technology Co. Ltd. (Shanghai, China). The data were analyzed on the free online platform of Majorbio I-Sanger Cloud Platform^1^. The raw reads of bacteria and fungi were deposited into the NCBI Sequence Read Archive (SRA) under the Bioproject number PRJNA509944 and PRJNA509988, respectively.

### Processing of Sequencing Data

Raw fastq files were demultiplexed, quality-filtered using Trimmomatic (Bolger et al., 2014) and merged using FLASH (Magoč and Salzberg, 2011) with the following criteria: (i) The reads were truncated at any site receiving an average quality score < 20 over a 50 bp sliding window. (ii) Primers were exactly matched, allowing two nucleotide mismatches, and reads containing ambiguous bases were removed. (iii) Sequences whose overlap was longer than 10 bp were merged according to their overlap sequence.

Subsequently, pairwise distances were calculated between all remaining unique sequences and a distance matrix was created. Operational taxonomic units (OTUs) were clustered using a 0.03 OTU definition (97% sequence similarity cut-off level) using UPARSE (version 7.1)^2^, and a majority consensus taxonomy was obtained for each OTU. To minimize the impact of sequencing artifacts, singletons were removed from the datasets (Dickie, 2010). Chimeric sequences were identified and removed using UCHIME (Edgar et al., 2011). The taxonomy of each 16S rRNA gene and ITS sequence was analyzed using the RDP Classifier algorithm^3^ against the Silva (SSU128) 16S rRNA database (bacteria) and UNITE v.7 ITS database (fungi) using a confidence threshold of 70%; the non-bacterial and non-fungal sequences were removed from the data sets.

### Soil Description and Analysis

Soil samples were first passed through a 1-mm mesh and the physicochemical properties were analyzed using the following methods: The pH was determined using a potentiometric method, and the total nitrogen and phosphorus contents of the soil were determined using the Kjeldahl method and the acid-solution-molybdenum antimony colorimetric method, respectively. The organic mass fraction was determined using the potassium dichromate capacity method (van Reeuwijk, 2006).

### Statistical Analysis

Statistical analyses were performed in R 3.5.1 (R Core Team, 2018). Significant differences in the variance of parameters were evaluated with ANOVA and Student’s *t*-test in SPSS 17.0. *Post hoc* comparisons were conducted by the Tukey’s honest significant differences tests. Student’s *t*-test was used to test the effect of the genetic modification, plant compartment (root, stem) and plant location (Daqing, Qiqihar) on the read abundances. Hierarchical clustering (based on Bray–Curtis dissimilarities) was performed with FastTree (version 2.1.3)^4^ and FastUniFrac (Hamady et al., 2010), and Principal co-ordinates analysis (PCoA) was performed in R. ANOSIM (an analog of univariate ANOVA) based on the Spearman_approx was used to statistically support the PCoA analyses. Indicator species analysis was performed using the multipat function of the indicspecies package in R (version 1.7.1) (De Cáceres and Legendre, 2009). Distance-based redundancy analysis (db-RDA) and Mantel test were used to analyze the relationship between microorganism and environmental factors. A taxonomic dendrogram (Figure 6, 7) was generated with one representative sequence of each OTU using FastTree and displayed with the use of iTOL (Interactive Tree Of Life) (Ivica and Peer, 2011).

## Results

### Quality Metrics of the Illumina Sequencing

Sequencing resulted in a total of 1,548,999 and 1,778,124 raw reads in the amplicon libraries of bacteria and fungi, respectively. The average read length before processing was 502 bp and 602 bp, respectively. After quality trimming and assigning reads to the different samples, 1,394,267 and 1,490,716 high quality reads remained in the bacteria and fungi datasets, with an average length (±standard deviation) of 395 bp ± 2 and 281 bp ± 5, respectively (Table 1A).

**Table 1:** Quality metrics of Illumina sequencing analysis.

(A) Total number of reads and read length before and after quality checking and trimming									
	
		Bacteria		Fungi					
Total of raw reads before QC		1548999		1778124					
Average read length before QC		502		602					
Total of assigned reads after QC		1394267		1490716					
Average read length after QC		395 ± 2		281 ± 5					

**(B) Assigned reads**		**Root**				**Stem**			
		
		**DAR**	**DBR**	**QAR**	**QBR**	**DAS**	**DBS**	**QAS**	**QBS**

Average of reads	Bacteria	53475 ± 1450	56322 ± 1404	48375 ± 3773	51063 ± 2249	67138 ± 6984	65134 ± 7121	62827 ± 9335	60422 ± 7691
	Fungi	53942 ± 3031	43887 ± 2355	51022 ± 4673	47400 ± 2332	74984 ± 3683	72160 ± 4296	81699 ± 3088	71812 ± 1953
Average read length	Bacteria	398 ± 1	397 ± 1	396 ± 1	395 ± 1	394 ± 0	394 ± 0	394 ± 0	394 ± 0
	Fungi	272 ± 9	272 ± 8	283 ± 35	288 ± 6	291 ± 11	252 ± 9	299 ± 11	293 ± 4

**(C) Non-target rRNA (%)**									

Mitochondria Chloroplast/plastid	Bacteria	0.05 ± 0.01	0.04 ± 0.01	0.02 ± 0.01	0.01 ± 0.01	0.02 ± 0.01	0.02 ± 0.01	0.03 ± 0.01	0.03 ± 0.01
	Fungi	0	0	0	0	0	0	0	0
	Bacteria	0	0	0	0	0	0	0	0
	Fungi	0	0	0	0	0	0	0	0

**(D) Unclassified reads**									

Reads (%)	Bacteria	0	0	0.03 ± 0.01	0	0.08 ± 0.04	0	0.36 ± 0.36	0
	Fungi	7.86 ± 3.99	4.80 ± 2.31	2.08 ± 1.34	1.04 ± 0.89	2.25 ± 0.98	11.38 ± 9.24	6.75 ± 3.26	8.38 ± 3.71


***(A)** Quality metrics before and after quality control (QC). The average read length was calculated based on 24 samples across all plant compartments. **(B)** The average number of assigned reads (±standard deviation) per plant compartment and the average length of assigned reads. Statistical differences at the 95% confidence interval are indicated with lowercase letters. DAR, Root of transgenic poplar in Daqing; DBR, Root of non-transgenic poplar in Daqing; QAR, Root of transgenic poplar in Qiqihar; QBR, Root of non-transgenic poplar in Qiqihar; DAS, Stem of transgenic poplar in Daqing; DBS, Stem of non-transgenic poplar in Daqing; QAS, Stem of transgenic poplar in Qiqihar; and QBS, Stem of non-transgenic poplar in Qiqihar. **(C)** Comparison of the number of non-target 16S rRNA and ITSs sequences (%) co-amplified during PCR amplification. **(D)** Reads that could not be unambiguously classified at the phylum level (“unclassified”) (%).*

We determined the co-amplification of non-target 16S rRNA and ITSs (chloroplast, plastid, and mitochondrial sequences), and then surveyed the number of reads that could not be unambiguously classified at the phylum level (Table 1). We found that, under our optimized PCR conditions, minute fractions of mitochondrial 16S rRNA, but no ITSs sequences, were co-amplified from root and stem samples from the two different locations (ranging from 0.01 to 0.05% of the normalized reads). No chloroplast, plastid, 16S rRNA, or ITSs sequences were co-amplified from any of the samples. In the bacterial and fungal amplicon sequences, we were able to classify the majority of reads, with only a relatively small proportion of reads remaining unclassified (ranging from 0 to 0.36% in bacteria, and 1.04 to 11.38% in fungi). Non-target and unclassified reads at the phylum level were removed from the dataset before further analysis (Table 1).

### Alpha Diversity

**FIGURE 1:**
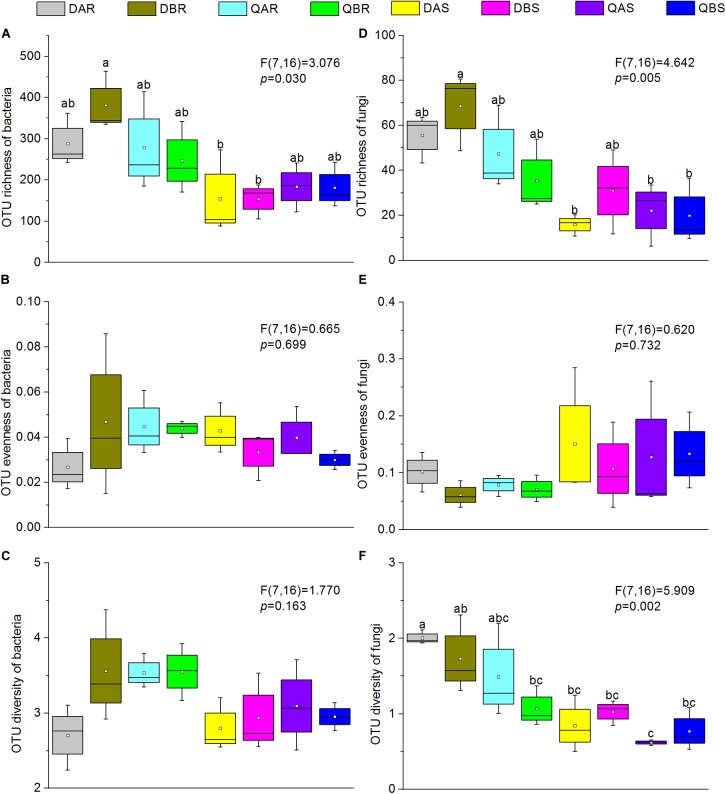
Alpha diversity estimates of bacterial and fungal communities in transgenic and non-transgenic poplars. **(A)** Operational taxonomic unit (OTU) richness of bacteria. **(B)** OTU evenness of bacteria. **(C)** OTU diversity of bacteria. **(D)** OTU richness of fungi. **(E)** OTU evenness of fungi. **(F)** OTU diversity of fungi. The richness, evenness, and diversity of bacterial and fungal communities are displayed with the bootstrap, Simpson evenness, and Shannon indices, respectively. Box plots display the first (25%) and third (75%) quartiles, the median, mean, and the maximum and minimum observed values within each data set. Alpha diversity estimates represent three biological replicates for each set. Data were analyzed using one-way analysis of variance (ANOVA) and Tukey post hoc comparisons. The overall plant compartment effects (F(DFn, DFd) and *p*-value) are displayed at the top of each graph. Significant differences (*p* < 0.05) across plant compartments are indicated using lowercase letters.

**FIGURE 2:**
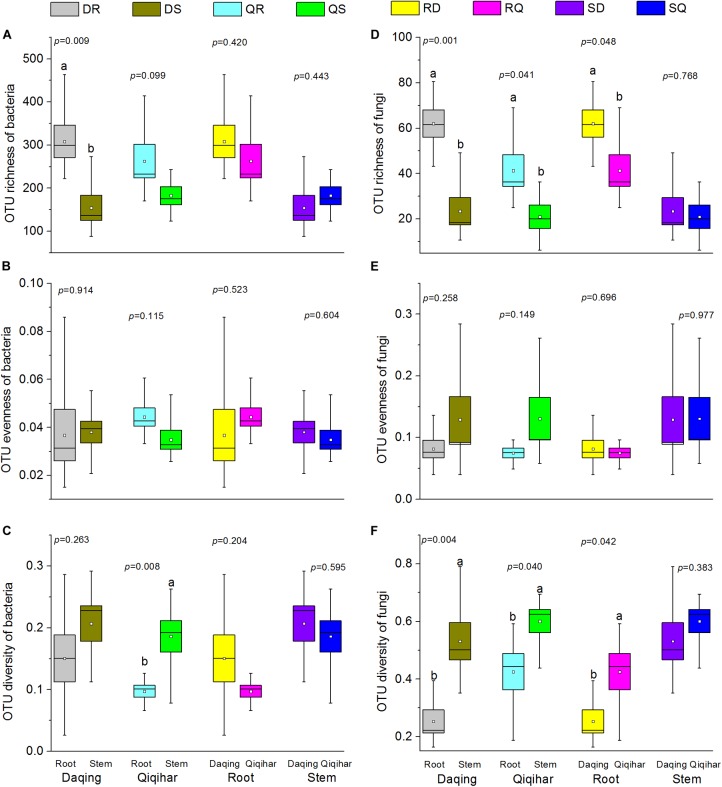
Alpha diversity estimates of bacterial and fungal communities in different environmental conditions and different tissues. **(A)** Operational taxonomic unit (OTU) richness of bacteria. **(B)** OTU evenness of bacteria. **(C)** OTU diversity of bacteria. **(D)** OTU richness of fungi. **(E)** OTU evenness of fungi. **(F)** OTU diversity of fungi. The richness, evenness, and diversity of bacterial and fungal communities are displayed with the bootstrap, simpsoneven, and Shannon indices, respectively. Box plots display the first (25%) and third (75%) quartiles, the median, mean, and the maximum and minimum observed values within each data set. Alpha diversity estimates represent three biological replicates for each set. Data were analyzed using Student’s *t-*test. The overall plant compartment effects (*p-*value) are displayed at the top of each graph. Significant differences (*p* < 0.05) across plant compartments are indicated using lowercase letters.

Alpha diversity, the microbial diversity within each sample, was analyzed based on the OTU richness, and the Simpson evenness and Shannon diversity index (Figure 1, 2). To control for differences in sampling effort across plant compartments, normalization was carried out according to the minimum sample sequence number before calculating the diversity indices. The reads of bacteria and fungi were standardized to 32,849 and 11,515, respectively. According to the 97% sequence similarity cut-off level, the reads of bacteria and fungi were clustered into 1493 and 321 OTUs, respectively. Rarefaction curves were assembled showing the numbers of OTUs and Shannon index, relative to the number of total sequences (Supplementary Figure S1).

The OTU richness, Simpson evenness, and Shannon diversity index of transgenic poplars were comparable with those of non-transgenic poplars (Figure 1). Except for the bacteria of Qiqihar, the bacteria and fungi OTU richness were highly dependent on the plant compartment, with higher richness values for root samples and decreased richness estimates in the stem samples (Figure 2A,D). The fungi OTU richness of the roots in Daqing was significantly higher than that of the roots from Qiqihar (Figure 2D). For evenness estimates, we found no clear separation between different plant compartments (root and stem) or between different locations (Daqing and Qiqihar) (Figure 2B,E). For the diversity estimates of bacteria, we found a clear separation between the root and stem in Qiqihar (Figure 1C). The same pattern appeared in the diversity estimate of fungi (Figure 2F). Higher fungal diversity measures were observed for the root compared with those in the stem in both Daqing and Qiqihar. Furthermore, the diversity of root endophytic fungi was higher in Daqing than in Qiqihar.

### Beta Diversity

**FIGURE 3:**
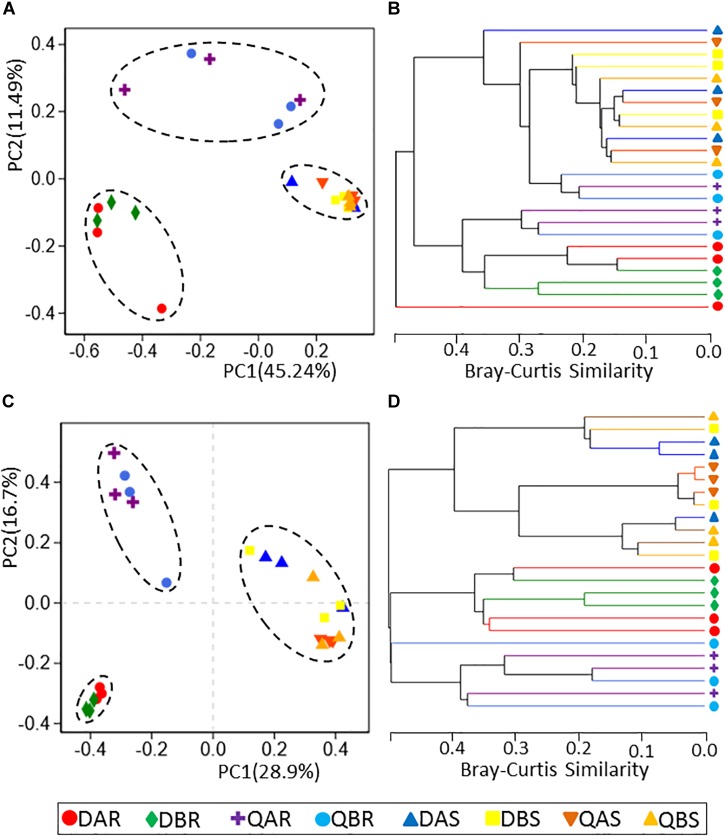
Principal co-ordinates analysis (PCoA) and hierarchical clustering of bacterial and fungal communities. **(A)** PCoA for the bacterial communities. **(B)** Hierarchical clustering for the bacterial communities. **(C)** PCoA for the fungal communities. **(D)** Hierarchical clustering for the fungal communities. PCoA of samples based on rarefaction to the minimum sample sequence number. Operational taxonomic units (OTUs) were defined at a 97% sequence similarity. Hierarchical clustering (group average linkage) of the samples based on Bray–Curtis dissimilarity. PCoA and hierarchical clusters analysis were based on three biological replicates.

To compare the microbial community composition and assess the differences between microbial communities, beta diversity was evaluated at two phylogenetic levels: The phylum level and the OTU level for bacteria, the class level and the OTU level for fungi. Overall similarities in bacterial and fungal communities’ structures among samples were displayed using principal co-ordinates analysis (PCoA). A hierarchical clustering tree was constructed based on Bray–Curtis dissimilarities for bacteria and fungi, respectively (Figure 3 and Supplementary Figure S2).

Principal co-ordinates analysis analyses revealed that the microbial communities were clustered by different plant tissues (root, stem) at each phylogenetic level (Figure 3A,C and Supplementary Figures S1A,C). The microbial communities of the roots from Daqing and Qiqihar were separated clearly, whether from transgenic or non-transgenic poplars; however, those of the stem could not be distinguished. At the OTU level, principal component (PC)1 explained 45.24% and PC2 explained 11.49% of the total variation for bacteria; and PC1 explained 28.9% and PC2 explained 16.7% of the total variation for fungi (Figure 3A,C). Moreover, the microbial communities of the transgenic or non-transgenic poplars could not be separated for either roots or stems. Hierarchical clustering of pairwise Bray–Curtis dissimilarities showed the same results (Figure 3B,D). Hierarchical clustering of fungi (at the OTU and phylum level) revealed complete clustering according to plant tissue for the root and stem samples (Figure 3D and Supplementary Figure S1D). To statistically support the results of the PCoA analyses and hierarchical clustering, analysis of similarity (ANOSIM), based on the Spearman_approx distance algorithm, was used to examine the samples of different plant compartments (Table 2). All results were similar to those of the above two analyses at the phylum, class and OTU level.

**Table 2:** Analysis of similarity (ANOSIM).

Microorganism	Effects factor	Phylogenetic level	OUT		Phylum/Class	
				
		ANOSIM output	*R*	*p*	*R*	*p*
Bacteria	Genetic modification	DAR vs. DBR	0.1111	0.201	-0.1852	0.718
		QAR vs. QBR	-0.1481	0.808	-0.1852	0.813
		DAS vs. DBS	-0.1111	1.000	0.0370	0.600
		QAS vs. QBS	-0.4074	1.000	0.0741	0.424
	Plant compartments	DR vs. DS	1	0.003**	0.2611	0.017*
		QR vs. QS	0.5537	0.003**	0.4944	0.002**
	Plant location	DR vs. QR	0.8254	0.001**	0.0944	0.158
		DS vs. QS	-0.0298	0.493	0.1074	0.116
Fungi	Genetic modification	DAR vs. DBR	-0.0370	0.791	0.3333	0.416
		QAR vs. QBR	-0.2222	1.000	-0.1296	1.000
		DAS vs. DBS	0.1481	0.201	0.3333	0.091
		QAS vs. QBS	0.0370	0.373	-0.0741	0.627
	Plant compartments	DR vs. DS	1	0.001**	1	0.003**
		QR vs. QS	0.9833	0.004**	0.9944	0.003**
	Plant location	DR vs. QR	0.9343	0.005**	0.6870	0.002**
		DS vs. QS	0.4204	0.021*	-0.0130	0.426


*Genetic modification, plant compartments, and plant location effects on the microbial community structures were calculated using analysis of similarities (ANOSIM) based on the Spearman_approx distance algorithm (999 permutations). Significance levels: ^∗^*p* ≤ 0.05; ^∗∗^*p* ≤ 0.01.*

### Microbial Communities Ordinations

**FIGURE 4:**
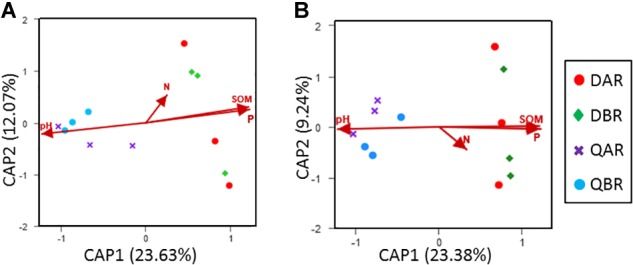
Distance-based redundancy analysis (db-RDA) of bacterial and fungal communities in root. **(A)** Db-RDA for the bacterial communities in the roots. **(B)** Db-RDA for the fungal communities in the roots. Db-RDA of samples based on Bray–Curtis dissimilarity and rarefaction to the minimum sample sequence number. Operational taxonomic units (OTUs) were defined at a 97% sequence similarity.

Correlations between microbial community structure and environmental factors were calculated to examine the environmental factors that could lead to variation in the microbial diversity (data of environmental factors are listed in Supplementary Table S1). Distance-based redundancy analysis (db-RDA) of bacterial and fungal communities in roots demonstrated that the samples were divided according to environmental factors in the plant location (Figure 4). The Mantel test results showed that pH and soil organic matter (SOM) contents significantly correlated with the microbial communities (*p* < 0.05). However, the nitrogen and phosphorus content did not appear to be important factors explaining the variance in the communities of poplar root endophytes (Table 3).

**Table 3:** Correlation between microbial communities and environmental factors.

Environment factors	Bacteria		Fungi	
		
	*R*	*p*	*R*	*p*
pH	0.2280	0.022*	0.3113	0.012*
SOM	0.2119	0.023*	0.3233	0.003**
N	-0.0871	0.669	0.0077	0.347
P	0.1904	0.070	-0.1594	0.908


*Correlations between microbial communities and environmental factors were calculated using the Mantel test based on the Bray–Curtis distance algorithm (999 permutations). The degree of correlation is shown as the *R* and *p-*value. Significance level: ^∗^*p* ≤ 0.05; ^∗∗^*p* ≤ 0.01.*

### Bacterial and Fungal Community Structure

**FIGURE 5:**
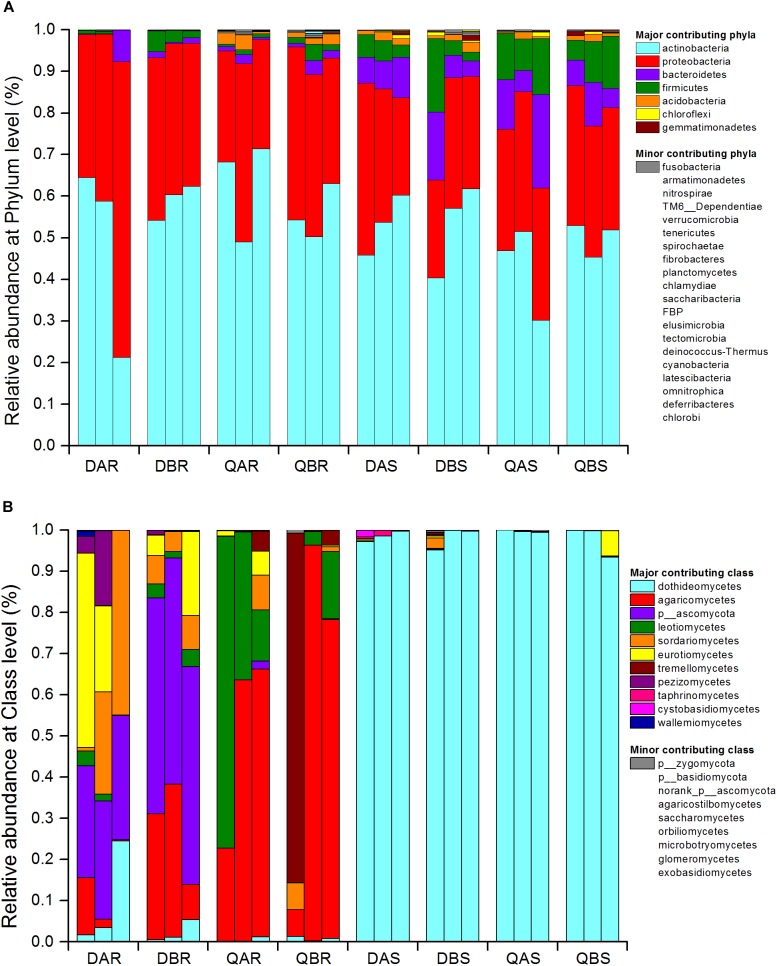
Bacterial community structure at the phylum level **(A)** and fungal community structure at the class level **(B)**. Relative sequence abundance of bacterial phyla associated with the root and stem endosphere. The three biological replicates are displayed in separate stacked bars. Major contributing phyla are displayed in different colors and minor contributing phyla are grouped and displayed in gray. The fungal class are represented in the same way. Total relative abundances of all phyla of bacteria and class of fungi and significant effects are listed in Supplementary Tables S2, S5, respectively.

At the level of the phylum and genus of bacteria, we evaluated all observed phyla and genera using Student’s *t*-test to test the effects of plant compartment (R vs. S), genetic modification (A vs. B), and plant location (D vs. Q) on their relative abundance (%). The bacterial community was dominated by actinobacteria (relative abundance around 50%), proteobacteria (around 35%), bacteroidetes (around 5%), and firmicutes (around 5%) (Figure 5 and Supplementary Table S2). For samples from different compartments, we observed a significant enrichment (*p* < 0.01) of *Rhodococcus* (relative abundance of root: 11.34%, stem: 42.13%), alcaligenaceae (root: 0.77%, stem: 3.31%), *Burkholderia-Paraburkholderia* (root: 0.86%, stem: 3.18%), *Ralstonia* (root: 1.75%, stem: 8.97%), *Bradyrhizobium* (root: 3.21%, stem: 0.34%), *Prevotella_9* (root: 0.03%, stem: 2.22%), *Sphingobium* (root: 1.06%, stem: 0.06%), *Streptomyces* (root: 19.84%, stem: 0.88%), and *Thauera* (root: 0.01%, stem: 2.04%) in roots compared to those in stems (Supplementary Table S3). To avoid the influence of the plant compartment, the comparison of root and stem samples from different locations were analyzed separately. For root samples from different locations, *Burkholderia-Paraburkholderia* (Daqing: 0.04%, Qiqihar: 1.68%), *Bosea* (Daqing: 2.25%, Qiqihar: 0.06%), *Ralstonia* (D: 0.26%, Q: 3.25%), *Variibacter* (D: 1.14%, Q: 0.05%), *Rhodococcus* (D: 2.36%, Q: 20.32%), alcaligenaceae (D: 0.11%, Q: 1.43%), *Variovorax* (D: 2.96%, Q: 0.41%), *Rhizobium* (D: 14.08%, Q: 1.05%), *Acidibacter* (D: 0.13%, Q: 3.37%), *Pseudonocardia* (D: 1.78%, Q: 0.30%), and *Phyllobacterium* (D: 1.11%, Q: 0.00%) were significantly enriched in Daqing and compared with that in Qiqihar (*p* < 0.05). For stem samples, only *Streptococcus* (D: 0.21%, Q: 0.76%) was significantly enrichment between the two sites (*p* < 0.01) (Supplementary Table S4). However, there was no significant difference observed between transgenic and non-transgenic poplars for either the root or stem samples (Supplementary Tables S2–S4). The total relative abundances of all phyla and genera significant effects across plant compartments, genetic modification, and plant location are listed in Supplementary Tables S2–S4.

At the class and genus level of fungi, all observed classes and genera were evaluated using ANOVA to test the effects of plant compartment (R vs. S), genetic modification (A vs. B), and plant location (D vs. Q) on their relative abundance (%). The fungal community of the roots was dominated by dothideomycetes, agaricomycetes, ascomycota, leotiomycetes, sordariomycetes, eurotiomycetes, and tremellomycetes. That of the stem was dominated by dothideomycetes (Figure 5 and Supplementary Table S5). A significant enrichment (*p* < 0.05) was observed in samples between the roots and stems at the genus level. Specifically for *Endosporium* and pleosporales, we observed an unusually high relative abundance in the stem (69.90 and 28.08%), but only 0 and 0.82% in the root, respectively. There were also marked distinctions for ascomycota (root: 20.73%, stem: 0.02%), thelephoraceae (root: 2.59%, stem: 0%), leotiomycetes (root: 0.84%, stem: 0%), and *Lachnum* (root: 0.50%%, stem: 0%) between roots and stems (Supplementary Table S6). Furthermore, the abundances of fungi belonging to ascomycota (Daqing: 41.07%, Qiqihar: 0.01%), leotiomycetes (D: 1.67%, Q: 0.01%), *Lachnum* (D: 0%, Q: 8.64%), and *Athelopsis* (D: 0%, Q: 44.21%) were significantly different between root samples from different plant locations. In addition, there was no significant difference between the stem samples from the two sites (Supplementary Table S7). Similar to bacteria, no significant difference was observed between transgenic and non-transgenic poplars at the class and genus level (Figure 5 and Supplementary Tables S5–S7). The total relative abundances of all classes significant effects across plant compartments, genetic modification, and plant location are listed in Supplementary Tables S5–S7.

**FIGURE 6:**
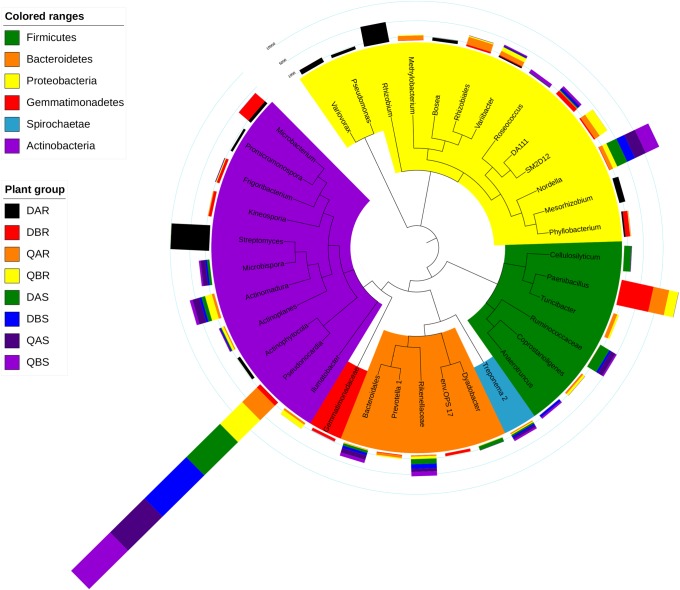
Top operational taxonomic units (OTU) members of the bacterial microbiome. A taxonomic dendrogram showing the core bacterial microbiome of each plant group. Color ranges identify phyla within the tree. Colored bars represent the relative abundance of each OTU in each plant group. The taxonomic dendrogram was generated with one representative sequence of each OTU using FastTree and displayed using iTOL (Interactive Tree Of Life). The total relative abundances of all OTUs and the significant effects across plant compartments are listed in Supplementary Table S8.

**FIGURE 7:**
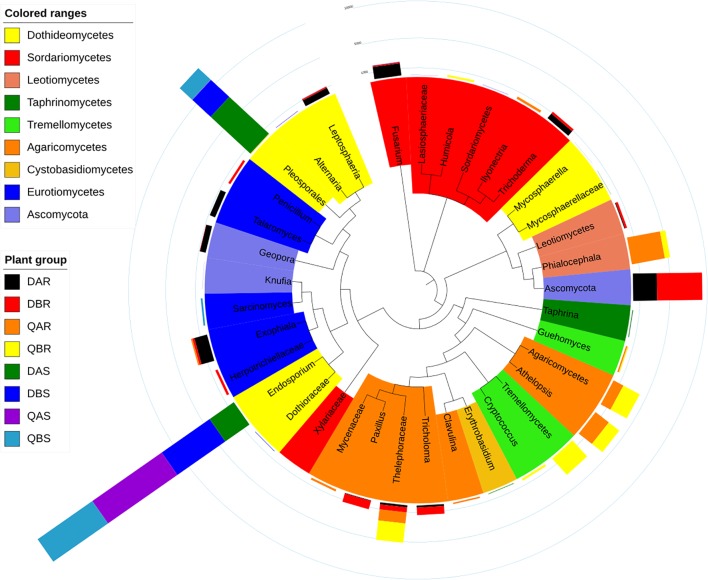
Top operational taxonomic units (OTU) members of the fungal microbiome. A taxonomic dendrogram showing the core fungal microbiome of each plant group. Color ranges identify classes within the tree. Colored bars represent the relative abundance of each OTU in each plant group. The taxonomic dendrogram was generated with one representative sequence of each OTU using FastTree and displayed using iTOL (Interactive Tree Of Life). The total relative abundances of all OTUs and the significant effects across plant compartments are listed in Supplementary Table S9.

For the OTUs, the top 10 most abundant OTUs of each group were defined as the core microbiome. Bacteria and fungi belonged to 37 OTUs representing 6 phyla and 36 OTUs representing 11 classes, respectively (Figure 6, 7 and Supplementary Tables S8, S9). The percentages of the core bacterial OTUs ranged from 59.18% to 68.22% in roots, and 68.80% to 75.91% in stems. We then tested the effect of plant compartment, genetic modification, and plant location on the OTU numbers of the core community members. ANOVA analysis showed significant (*p* < 0.05) effects of plant compartment, genetic modification, and plant location in 14 (37.84%), 3 (8.11%), and 7 (18.92%) core bacterial OTUs, respectively. In the stem samples, *Pseudonocardia* (36.63–45.52%), *Nordella* (6.66–11.48%), rikenellaceae (2.40–3.98%), bacteroidales (1.87–3.59%), and *Treponema_2* (1.14–1.55%) were significantly enriched (*p* < 0.01) compared with those in the root samples. For the effects of plant location, *Nordella* (2.83–11.48%), *Actinomadura* (1.78–5.48%), *Variibacter* (0.73–3.42%), bacteroidales (0.36–3.59%), and *Treponema_2* (0.76–1.55%) were significantly enriched (*p* < 0.05) in the samples of Qiqihar compared with those from Daqing. Finally, we found a significant enrichment (*p* < 0.05) of *Frigoribacterium* (0.15–2.02%), DA111 (0.25–3.45%), and *Anaerotruncus* (0.06–1.76%) between the transgenic and non-transgenic poplars (Figure 6). The total relative abundances of all core OTUs and the significant effects are listed in Supplementary Table S8.

The percentages of the core fungal OTUs ranged from 92.22 to 97.92% in roots, and 98.80 to 99.86% in stems. We tested the effect of plant compartment, genetic modification, and plant location on the OTU numbers of the core community members. ANOVA analysis (*p* < 0.05) showed significant effects of plant compartment, genetic modification, and plant location in 6 (16.67%), 0, and 7 (8.33%) core fungal OTUs, respectively. Ascomycota (0–52.77%), thelephoraceae (2.44–22.74%), and *Athelopsis* (0–19.52%) were significantly enriched (*p* < 0.05) in the root samples but not in the stem samples. By contrast, pleosporales (0.41–63.14%) was enriched in stems. In particular *Endosporium* (34.50–98.90%) appeared in large numbers in stems, but was not found in the root samples. Furthermore, in the root samples, we observed a significant difference (*p* < 0.05) in the abundances of ascomycota, leotiomycetes, and *Athelopsis* between Daqing and Qiqihar (Figure 7). Finally, the total relative abundances of all core OTUs and the significant effects are listed in Supplementary Table S9.

To ascertain which OTUs are responsible for the observed community structure differentiation between the different groups, species indicator analyses were used to reveal significant associations between OTUs and plant compartments, genetic modification events, and plant locations. Indicator analyses were performed on full community matrices. The full lists of indicator OTUs and their corresponding indicator values can be found in Supplementary Tables S10, S11.

Species indicator analysis of bacteria revealed 90 indicator OTUs in the root samples, 39 in the stems, 27 in the samples from Daqing, 19 in the samples from Qiqihar, 2 in the transgenic poplar, and 5 in non-transgenic poplar (see Supplementary Table S10). However, when we calculated the indicator OTUs of bacteria with an average abundance of more than 1%, we found 14 indicator OTUs in the root samples [*Streptomyces*, *Rhizobium*, *Mycobacterium*, *Sphingobium*, *Bradyrhizobium*, *Mesorhizobium*, micromonosporaceae, *Actinoplanes*, *Acidibacter*, *Variovorax*, *Actinophytocola*, *Pseudonocardia*, *Amycolatopsis* and *Bosea* (*p* < 0.01)], 5 in the stem samples [*Thauera*, *Rhodococcus*, *Prevotella_9*, *Burkholderia* (*p* < 0.01), and geodermatophilaceae (*p* < 0.05)], 3 in the samples from Daqing [*Rhizobium* (*p* < 0.01), *Mesorhizobium*, and *Actinophytocola* (*p* < 0.05)], and 1 in the samples from Qiqihar (*Acidibacter*; *p* < 0.05) (Table 4). No indicator OTU greater than 1% was found between transgenic and non-transgenic poplars.

**Table 4:** Indicator species analysis of bacteria.

OTU (Genus	Associated	Indicator		Relative
or higher)	with	value	*p*-Value	abundance(%)
*Streptomyces*	Root	0.999	0.001**	17.56
*Rhizobium*	Root	0.999	0.001**	7.55
*Mycobacterium*	Root	0.987	0.001**	4.03
*Sphingobium*	Root	0.978	0.001**	1.06
*Bradyrhizobium*	Root	0.973	0.001**	3.10
*Mesorhizobium*	Root	0.965	0.001**	2.10
micromonosporaceae	Root	0.957	0.001**	4.10
*Actinoplanes*	Root	0.957	0.001**	2.85
*Acidibacter*	Root	0.957	0.001**	1.73
*Variovorax*	Root	0.956	0.001**	1.69
*Bosea*	Root	0.895	0.004**	1.15
*Actinophytocola*	Root	0.866	0.001**	3.06
Pseudonocardia	Root	0.866	0.001**	1.00
*Amycolatopsis*	Root	0.865	0.001**	1.37
*Thauera*	Stem	0.996	0.001**	2.04
*Rhodococcus*	Stem	0.977	0.001**	1.15
*Prevotella_9*	Stem	0.950	0.001**	2.22
*Burkholderia*	Stem	0.941	0.001**	1.52
Geodermatophilaceae	Stem	0.907	0.020*	1.68
*Rhizobium*	Daqing	0.764	0.007**	4.12
*Mesorhizobium*	Daqing	0.707	0.032*	1.60
*Actinophytocola*	Daqing	0.705	0.047*	3.04
*Acidibacter*	Qiqihar	0.762	0.035*	1.58


*Associations were calculated using species indicator analyses (Indicator value) in R. Data table shows the results for the analysis where rare operational taxonomic units (OTUs) (<1% relative abundance) were excluded. Significance levels: ^∗^*p* ≤ 0.05; ^∗∗^*p* ≤ 0.01. Full results of the indicator species analysis are presented in Supplementary Table S10.*

Meanwhile, species indicator analysis of fungi revealed 23 indicator OTUs in the root samples, 2 in the stem samples, 8 in the samples from Daqing, and 8 in the samples from Qiqihar; no indicator OTUs were found between the transgenic and non-transgenic poplars (see Supplementary Table S11). For the indicator OTUs with an average abundance of more than 1%, nine OTUs were found in root samples [*Exophiala*, ascomycota, *Fusarium*, leptosphaeria (*p* < 0.01), tremellomycetes, thelephoraceae, *Phialocephala*, trechisporales, tricholoma (*p* < 0.05)], two in stem samples [*Endosporium*, pleosporales (*p* < 0.01)], three in the samples from Daqing [*Fusarium*, thelephoraceae, tricholoma (*p* < 0.05)], and three in the samples from Qiqihar [tremellomycetes (*p* < 0.01), *Phialocephala*, trechisporales (*p* < 0.05)] (Table 5).

**Table 5:** Indicator species analysis of fungi.

OTU (Genus	Associated	Indicator		Relative
or higher)	with	value	*p*-Value	abundance(%)
*Exophiala*	Root	0.913	0.001**	4.90
Ascomycota	Root	0.864	0.001**	20.54
*Fusarium*	Root	0.859	0.008**	2.18
Leptosphaeria	Root	0.816	0.002**	2.34
Tremellomycetes	Root	0.707	0.015*	6.37
Thelephoraceae	Root	0.707	0.012*	2.09
*Phialocephala*	Root	0.645	0.038*	11.35
Trechisporales	Root	0.645	0.036*	6.09
Tricholoma	Root	0.645	0.029*	2.79
*Endosporium*	Stem	1.000	0.001**	69.90
Pleosporales	Stem	0.996	0.002**	28.08
*Fusarium*	Daqing	0.813	0.017*	2.37
Thelephoraceae	Daqing	0.707	0.013*	2.09
Tricholoma	Daqing	0.645	0.031*	2.79
Tremellomycetes	Qiqihar	0.707	0.008**	6.37
*Phialocephala*	Qiqihar	0.645	0.035*	11.35
Trechisporales	Qiqihar	0.645	0.022*	6.09


*Associations were calculated using species indicator analyses (Indicator value) in R. Data table shows the results for the analysis where rare operational taxonomic units (OTUs) (<1% relative abundance) were excluded. Significance levels: ^∗^*p* ≤ 0.05; ^∗∗^*p* ≤ 0.01. Full results of the indicator species analysis are presented in Supplementary Table S11.*

**FIGURE 8:**
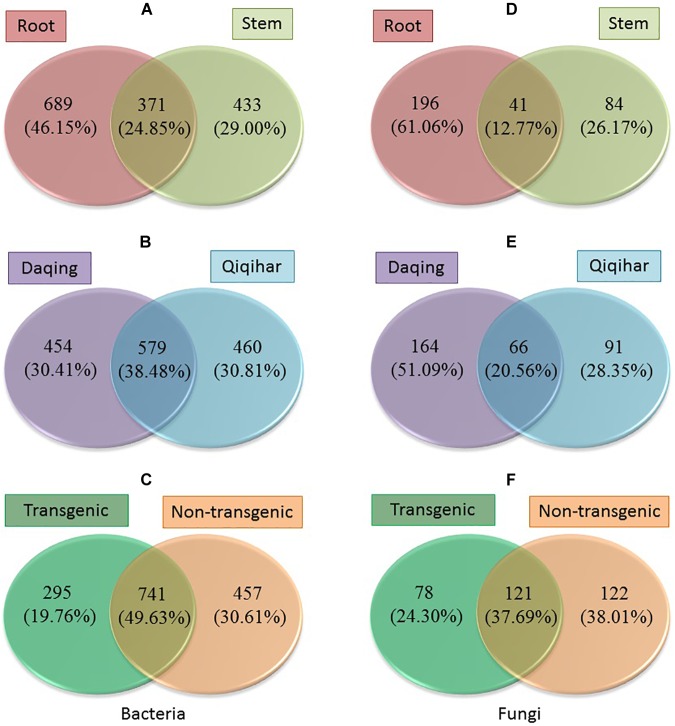
Operational taxonomic units (OTUs) distribution within different groups of bacteria and fungi. **(A–C)** Number and proportion of OTUs of bacteria in different groups. **(D–F)** Number and proportion of OTUs of fungi in different groups.

The number and proportion of OTUs of bacteria and fungi in each group and shared by different groups were calculated to give an overview of the OTU distribution within the different groups (Figure 8). For bacteria, the numbers of OTUs in the root and stem samples were 689 (46.15%) and 433 (29.00%), and they shared a relatively small amount of OTUs (only about 25%; Figure 8A). Approximately 30% of OTUs were found in the samples of both Daqing and Qiqihar, and they shared about 38% of all OTUs (Figure 8B). Transgenic and non-transgenic poplar shared about half of the OTUs (49.63%) (Figure 8C). The same pattern was found in fungi, for which we clearly observed a higher overlap in OTUs from the transgenic poplar and non-transgenic poplar (37.69%) compared with that in the roots and stems (12.77%) and Daqing and Qiqihar (20.56%) (Figure 8D).

## Discussion

### Did Genetic Modification Change the Microbial Community in Poplar?

Several studies in transgenic maize on the effect of the diversity and population size of microbial endogenous communities have revealed that they were not significantly affected by transformation of maize with *Bacillus thuringiensis* toxin (Bt) (Prischl et al., 2012; Silva et al., 2014; Sun et al., 2017). Similarly, based on the alpha diversity, we found that the OTU richness, evenness, and diversity of bacteria and fungi in transgenic poplars were comparable with those of non-transgenic poplars (Figure 1). The beta diversity and community structure analyses (Figure 3, 4) results exhibited the same pattern. Transgenic and non-transgenic poplars were not clearly distinguished, and no indicator OTUs greater than 1% were found between transgenic and non-transgenic poplars (Tables 3, 4). The proportion of OTUs shared by transgenic and non-transgenic poplars were 49.63% and 37.69% for bacteria and fungi, respectively (Figure 8C,F). These results indicated that the genetic modification events did not affect the diversity and structure of the endogenous bacterial and fungal communities in the aboveground and underground parts of poplar trees. The expression of resistance genes in transgenic bananas had no consequences for non-target rhizobacteria and endophytes (Nimusiima et al., 2015). However, another study found that the presence of transgenes was a factor for the changes detected in the endophytic fungal community of maize leaves (Silva et al., 2016). The root interior microbial communities of transgenic canola were less diverse, but differed from those in non-transgenic plants (Dunfield and Germida, 2001; Siciliano and Germida, 2010). Transgenic tobacco also had altered rhizosphere/rhizoplane microbial communities; however, these effects were temporary, and the diversity of the community was restored to the original level after one cycle of plant cultivation (Andreote et al., 2008). Previous research in our laboratory found no significant differences in bacterial communities between rhizosphere soils of 8-year-old GM and non-GM poplars (*Populus* × *euramericana* ‘*Guariento*’) (Zhu et al., 2016). Our transgenic poplar is different from most transgenic plants in that the exogenous gene, the *JERF* transcription factor, does not contain the *Bt* gene, and does not release the BT protein into the plant body and surrounding soil. Currently, our transgenic poplar has been tested for about 10 years, and the endophytic microbial communities of the roots and stems have not been affected. Poplar is a perennial woody plant, and the genetic modification effect on the environment is a long - term process.

### How Variable Are Microbial Communities of Poplars Grown in Saline and Non-saline Sites?

Environmental factors, such as soil quality, are the primary drivers of the plant endophytic microbiome (Podolich et al., 2014; Whitaker et al., 2018). A number of studies have shown that soil salinity, pH, and temperature may significantly influence the endophytic microbial community structure (Lozupone and Knight, 2007; Yaish et al., 2016b; Thiem et al., 2018). A recent study of the associations of root-inhabiting fungi in 19 herbaceous plant species, together with soil chemical properties, revealed that the phosphorus contents in soils correlated negatively with the intensity of mycorrhizal colonization (Rozek et al., 2018). However, the effect of pH on endophytic communities is largely unexplored. Most of the studies on endophytes have focused on herbaceous plant species (Kesari et al., 2013; Rozek et al., 2018).

In general, we found that the microbial communities of roots from Daqing and Qiqihar were clearly separated for both transgenic or non-transgenic poplars; however, those of the stem could not be distinguished (Figure 2). Such results have also been observed in *Alnus glutinosa* (Thiem et al., 2018) and *Phoenix dactylifera* (Yaish et al., 2016a). Our results showed that within the endophytic communities, the effects of pH and SOM contents were significant across both genotypes and regions for both bacterial and fungal communities (Figure 4 and Table 3). Our conclusions are in agreement with those of previous studies (Marschner et al., 2005; Ishida et al., 2009; Lauber et al., 2009; Shakya et al., 2013). In particular, Hartman et al. (2008) found that pH was the best predictor of changes in soil bacterial communities, and they observed changes in phylum-level abundances across the pH gradient for acidobacteria and actinobacteria. A study in a boreal forest along an 80-m-long successional transect located on the land-uplift coast showed a strong directional relationship between the organic matter characteristics and structure of the vegetation and microbial communities along the study transect (Merilä et al., 2010).

Endophytic bacteria in *Populus* roots were dominated by gammaproteobacteria and alphaproteobacteria (Gottel et al., 2011), and acidobacteria were also present at a higher level (Shakya et al., 2013), which were similar to our results (Figure 5). Moreover, our studies showed that the alphaproteobacteria and betaproteobacteria were found more frequently at the saline-alkali site (Daqing), which is related to plant resistance to environmental stress (Gołȩbiewski et al., 2014; Thiem et al., 2018). In the present study, the non-saline-alkali site (Qiqihar) had a higher abundance of gammaproteobacteria compared with that of the saline-alkali site (Daqing). This may be related to the higher abundance of SOM in the saline-alkali site (Shakya et al., 2013).

Bacteria belonging to *Bosea*, *Variibacter*, *Rhizobium*, *Variovorax*, *Pseudonocardia*, and *Phyllobacterium* were found more frequently in the root samples at the saline-alkali site (Daqing) (Supplementary Table S2). *Bosea* is a genus of bacteria in the bradyrhizobiaceae family, which has the ability to remove and degrade the pollutants arsenic, antinomy, and ciprofloxacin (Lu et al., 2018; Zhang et al., 2018). The genus *Variibacter* was first proposed by Kim et al. (2014). One of its members, *V. gotjawalensis* GJW-30^T^, was sequenced by Lee et al. (2016), who found that various genes of strain GJW-30^T^ encode functional enzymes for nitrate reduction, ginsenoside biosynthesis and degradation, and gibberellin biosynthesis and inactivation. The presence of these genes indicate an interaction relationship with plants. Members of *Rhizobium* are common microsymbionts of nodulating legumes (Azarias Guimarães et al., 2015). However, these symbiotic bacteria have been observed on non-legumes, such as maize, rice, and oats plants (Antoun et al., 1998). One of its members (*R. metallidurans*) was also isolated from roots of silver birch and alder growing on heavy metal-contaminated sites (Zloch et al., 2016). Strains belonging to the genus *Variovorax* have been demonstrated to degrade a broad range of different compounds, including pesticides and herbicides (Sorensen et al., 2008). However, bacteria belonging to *Burkholderia-Paraburkholderia*, *Ralstonia*, *Rhodococcus*, alcaligenaceae, and *Acidibacter* were found more frequently in the root samples at the non-saline-alkali site (Qiqihar) (Supplementary Table S3). Among them, *Burkholderia-Paraburkholderia*, *Ralstonia*, and alcaligenaceae belong to the burkholderiaceae, which contribute to soil suppressiveness via the production of sulfurous antifungal volatile organic compounds (Carrion et al., 2018).

In the present study, the endophytic fungal community of poplar tree roots was dominated by dothideomycetes, agaricomycetes, ascomycota, leotiomycetes, sordariomycetes, eurotiomycetes, and tremellomycetes. The stem samples were dominated by dothideomycetes (Figure 5). Our results were in agreement with those of a previous study in *Populus deltoides* (Shakya et al., 2013). We observed a lower abundance of fungi belonging to ascomycota and leotiomycetes at the non-saline-alkali site (Qiqihar) (Supplementary Table S7). Studies have shown that high levels of phosphorus in soil can reduce the abundance of ectomycorrhizal fungi in the ascomycota and leotiomycetes (Balzergue et al., 2013; Eva et al., 2015). Furthermore, *Lachnum* and *Athelopsis* were more abundant at the non-saline-alkali site (Qiqihar). *Lachnum* is commonly found in many plants, such as *Rubus*, *Quercus*, fern, bamboo, *Rosa*, and *Juglans* from diverse regions of the world (Ye et al., 2006).

### What Is the Difference in the Microbial Communities Between the Aboveground and Underground Parts of Poplar?

To compare the endophytic communities present in the plant compartments, we estimated the alpha diversity, focusing on OTU richness, evenness, and diversity (Figure 1). We found that the estimated richness was dependent on plant tissues, and that roots had higher OTU richness than stems. These results were consistent with the general view of endogenous colonization. There are highly rich and diverse rhizosphere microbiomes in soil, and some soil-borne bacteria could actively or passively pass the endodermis and pericycle, and reach the xylem vessels, ultimately leading to systemic colonization of the plant (Hardoim et al., 2008; Compant et al., 2009). The bacterial and fungal OTUs shared by the root and stems were 24.85 and 12.77%, respectively (Figure 7). PCoA and hierarchical clustering analysis of the bacterial and fungal communities were used to compare the bacterial and fungal community structures (Figure 2). For bacteria, all samples were strongly clustered according to plant compartment at the phylum and OTU levels. For fungi, all samples were strongly clustered according to plant compartment at the class and genus levels. The same niche differentiation between root, stem, and leaves has been described in poplar (*Populus tremula* × *Populus alba*), cacti, and willow (Fonseca-García et al., 2016; Tardif et al., 2016; Beckers et al., 2017). The relevant biotic and abiotic gradients, such as availability of soluble organic compounds, exist in plant microenvironments or plant tissues (root and stem) (Bulgarelli et al., 2013). In our study, there were 46.15% and 29% unique bacterial OTUs in the root and stem, and 61.06 and 26.17% unique fungal OTUs in the root and stem samples, respectively.

At the phylum level, actinobacteria and proteobacteria dominated the endophytic bacterial communities in the roots and stems (Figure 4). Actinobacteria have been reported to colonize any tissue or organ of the host plant (Dinesh et al., 2017). Different tissues and organs of the plant can be colonized by different actinobacteria, which might be determined by host-microbe interactions (Nimnoi et al., 2010). We found that the abundance of actinobacteria was higher in the roots than in stems (Figure 4). In addition, there is evidence that endophytic actinobacteria are abundant in roots, but occur moderately in the stems (Madhurama et al., 2014). Proteobacteria were also found to be abundant in underground and aboveground tissues of plants (Beckers et al., 2017; Wallace et al., 2018). However, bacteria belonging to bacteroidetes and firmicutes were more abundant in stems. This in agreement with the studies of (a) Jin et al. (2014), who revealed that Bacteroidetes existed in stems and leaves, but were absent in root samples; and (b) Yang et al. (2017), who found that sequences assigned to firmicutes (14.30%) and bacteroidetes (5.90%) in the leaf endophytic communities were more abundant compared with that in the root samples.

At the genus level, the root endophytic bacterial communities were dominated by *Streptomyces*, *Bradyrhizobium*, and *Rhizobium*. Dominant members of the stem samples were *Rhodococcus*, *Burkholderia-Paraburkholderia*, alcaligenaceae, *Ralstonia*, and *Prevotella_9.* These genera have been found in many plants, and may be beneficial for plant health and growth (Ulrich et al., 2008; Yu et al., 2015; Liu et al., 2017). Such a distribution pattern for the endophytic *Streptomyces*, *Bradyrhizobium*, and *Rhizobium* seems rational because the roots have maximum exposure to, and interactions with, the microbial population in the rhizosphere (Singh and Dubey, 2018). Studies have shown that, *R. fascians*, a member of *Rhodococcus*, and a teratogenic phytopathogen, is generally associated with the production of cytokinins, but is also able to produce the auxin indole acetic acid (IAA) via the indole-3-pyruvic acid (IPyA) pathway (Vandeputte et al., 2005). In the genomes of the burkholderiaceae, there are up to 30 genes for aromatic compound degradation, which suggested a suitable approach and microbes for the degradation of organic pollutants in the environment (Perez-Pantoja et al., 2012; Lunsmann et al., 2016).

Finally, for the endophytic fungal communities, at the OTU level (genus or higher), thelephoraceae, leotiomycetes, and *Lachnum* were dominant in the roots, and the stem samples were dominated by *Endosporium* and pleosporales. Thelephoraceae is a member of thelephorales, and all fungi within the order are ectomycorrhizal, forming mutually beneficial associations with the roots of living trees (Hibbett, 2007). Leotiomycetes comprise many ectomycorrhizal representatives, and they are abundant in the soil and plant roots (Thiem et al., 2018). *Endosporium* belong to Myriangales, which were also isolated from buds and twigs of *Populus* (Tsuneda et al., 2000, 2008).

## Conclusion

We showed that transgenic events did not affect the endophytic bacterial and fungal diversity of poplar (*Populus alba* × *P. berolinensis*). Bacterial and fungal community structure depends on pH and SOM content; however, the nitrogen and phosphorus content did not appear to be important factors explaining the variance in the communities of poplar root endophytes. The microbial communities appear to be stable in the stem, even under different environmental conditions. Furthermore, our data confirmed the microbiome niche differentiation at the root and stem compartments. Each plant compartment represents a unique ecological niche for the microbial communities. Finally, we identified the indicator OTUs and core microbiome associated with the different ecological niches of *Populus* and different environmental conditions. This may provide a basis for further study of host-microbial interactions using the identified abundant OTUs of *Populus*.

## Author Contributions

YW and WZ contributed to samplings, data analysis, and writing the manuscript. XS, CD, BZ, QH and RH were involved in devising and directing the experiments, and proofreading the manuscript. XS contributed to the concept of the research, gave constructive advice on the experiments, and finally completed the manuscript. All authors reviewed the manuscript and agreed to the publication of this manuscript.

## Conflict of Interest Statement

The authors declare that the research was conducted in the absence of any commercial or financial relationships that could be construed as a potential conflict of interest.

## Footnotes

1 www.i-sanger.com

2 http://drive5.com/uparse/

3 http://rdp.cme.msu.edu/

4 http://www.microbesonline.org/fasttree/
