# Supplementary figures and images for: Endophytic Communities of Transgenic Poplar Were Determined by the Environment and Niche Rather Than by Transgenic Events
Source: Front Microbiol. 2019 Mar 26;10:588. doi: 10.3389/fmicb.2019.00588 (PMC6445066; doi:10.3389/fmicb.2019.00588)

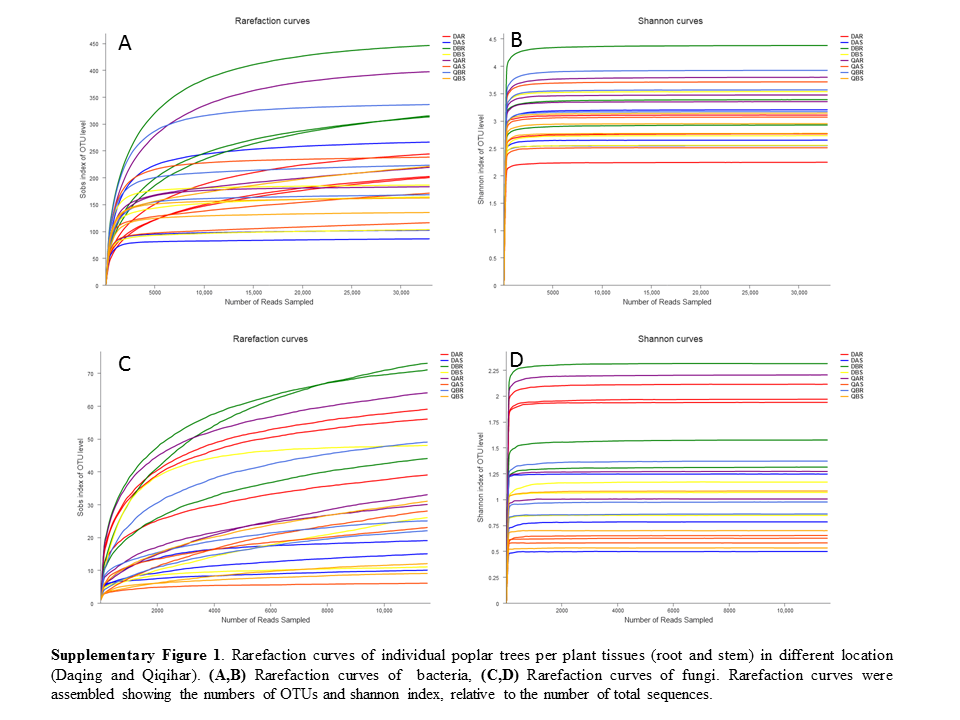

Supplement: Supplementary file 12 [file Image_1.TIF]

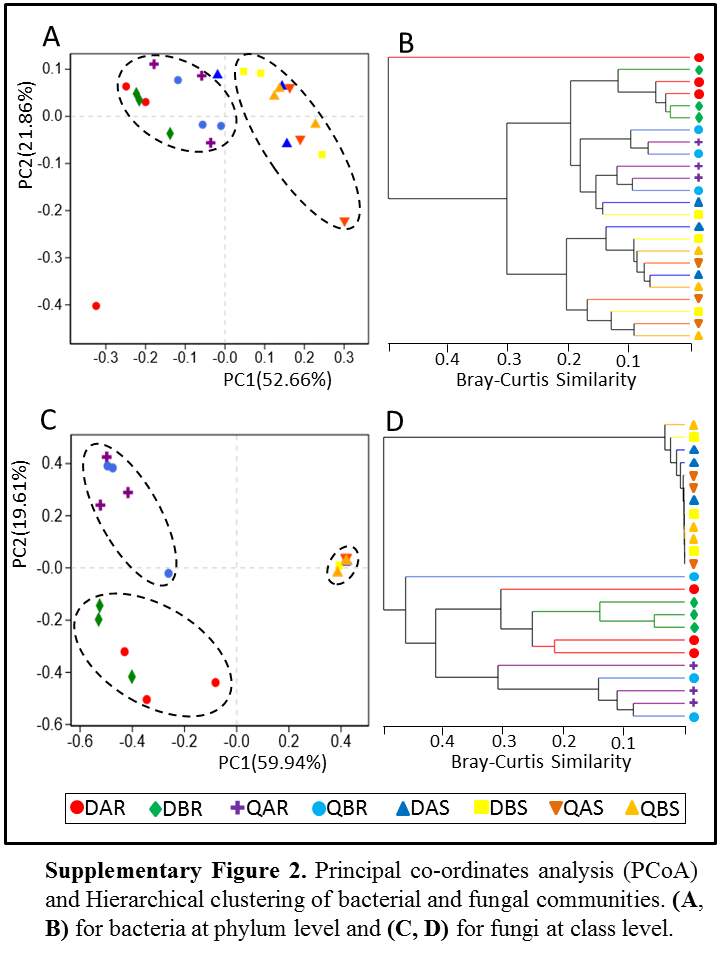

Supplement: Supplementary file 13 [file Image_2.TIF]
